# Active bone marrow S-values for the low-energy electron emitter terbium-161 compared to S-values for lutetium-177 and yttrium-90

**DOI:** 10.1186/s40658-022-00495-7

**Published:** 2022-09-24

**Authors:** Jens Hemmingsson, Johanna Svensson, Nicholas P. van der Meulen, Cristina Müller, Peter Bernhardt

**Affiliations:** 1 Department of Medical Radiation Sciences, The Sahlgrenska Academy, Sahlgrenska University Hospital, Gula Stråket 2B, 41345 Gothenburg, Sweden; 2 Department of Oncology, The Sahlgrenska Academy, Sahlgrenska University Hospital, Gothenburg, Sweden; 3 Center for Radiopharmaceutical Sciences, Paul Scherrer Institute, 5232 Villigen, Switzerland; 4 Laboratory of Radiochemistry, Paul Scherrer Institute, 5232 Villigen, Switzerland; 5 Department of Chemistry and Applied Biosciences, ETH Zurich, 8093 Zurich, Switzerland

**Keywords:** Dosimetry, Bone marrow, Terbium-161, Lutetium-177, Yttrium-90

## Abstract

**Background:**

Based on theoretical and preclinical results, terbium-161 may be a valid alternative to lutetium-177 and yttrium-90 in radionuclide therapies. The large low-energy electron emission from terbium-161 is a favorable feature in the treatment of disseminated disease, but its impact on the radiosensitive bone marrow needs to be evaluated. Using voxel-based skeletal dosimetry models in which active bone marrow is defined as regions containing stem cells and progenitor cells of the hematopoietic lineage, we generated S-values (absorbed dose per decay) for terbium-161 and evaluated its distribution-dependence in bone marrow cavities.

**Methods:**

S-values in the active bone marrow were calculated for terbium-161, lutetium-177, and yttrium-90 irradiation using two (male/female) image-based bone marrow dosimetry models. The radionuclides were distributed to one of the three structures that define the spongiosa bone region in the skeletal models: (i) active bone marrow, (ii) inactive bone marrow, or (iii) surface or whole volume of the trabecular bone. Decay data from ICRP 107 were combined with specific absorbed fractions to calculate S-values for 13 skeletal sites. To increase the utility, the skeletal site-specific S-values were averaged to produce whole-body average S-values and spongiosa average S-values.

**Results:**

For yttrium-90, the high-energy *β* particles irradiate the active marrow regardless of the source compartment, consistently generating the highest S-values (65–90% higher). Between terbium-161 and lutetium-177, the largest differences in S-values were with an active marrow source (50%), such as self-irradiation, due to the contribution of the short-ranged conversion and Auger electrons from terbium-161. Their influence decreased as the source moved to inactive marrow or the surface or volume of the trabecular bone, reducing the S-values and the differences between terbium-161 and lutetium-177 (15–35%).

**Conclusion:**

The S-values of terbium-161 for active bone marrow and, consequently, the bone marrow toxicity profile were more dependent on the radionuclide distribution within the bone marrow cavity than the S-values of lutetium-177 and yttrium-90. This effect was attributed to the considerable low-energy electron emission of terbium-161. Therefore, it will be critical to investigate the bone marrow distribution of a particular radiopharmaceutical for accurate estimation of the active bone marrow dose.

**Supplementary Information:**

The online version contains supplementary material available at 10.1186/s40658-022-00495-7.

## Background

Peptide receptor radionuclide therapy (PRRT) of neuroendocrine tumors (NETs) with ^177^Lu-labeled somatostatin analogues, such as [^177^Lu]Lu-DOTATATE, is generally considered safe, with the primary risk organs being the bone marrow and kidneys [1, 2]. The kidneys are mainly irradiated through an active uptake process, such as active reabsorption of [^177^Lu]Lu-DOTATATE in the proximal tubules, and the bone marrow is assumed to be irradiated either by circulating radiopeptides in the blood or through an intrinsic bone marrow uptake [3, 4]. Commonly observed toxicities after [^177^Lu]Lu-DOTATATE treatments refer mainly to the hematological tissue [5, 6]. In addition, long-term effects, such as leukemia and myelodysplastic disorder, are seen in 1–2% of patients [1, 7].

In a study of 807 patients with NETs, Bodei et al. reported that patients who received [^90^Y]Y-octreotide suffered from higher rates of kidney and hematological toxicities than those who received [^177^Lu]Lu-DOTATATE [7]. This is probably associated with the differences in emission characteristics, as yttrium-90 is a long-range pure *β*¯ emitter with a mean soft tissue range of 2.4 mm compared to 0.23 mm for the *β*¯ particles of lutetium-177. Recently, the *β*¯ particle emitter terbium-161 was proposed as an alternative to lutetium-177 in PRRT [8]. In addition to the chemical similarities between these two radiolanthanides that facilitate the use of existing targeting agents, terbium-161 emits 12.4 conversion and Auger electrons below 50 keV per decay compared to only 1.2 in the case of lutetium-177 (Table 1) [9]. This is considered a desirable feature in targeted radionuclide therapies because the ionization density of the emitted electrons is increased at lower energies [10–14]. A number of theoretical and preclinical studies have suggested superior tumoricidal properties of terbium-161 compared to other radionuclides [15–18]. Furthermore, the safety and imaging capabilities were demonstrated using [^161^Tb]Tb-DOTATOC at low activity in a clinical first-in-human study with two NET patients [19].

**Table 1:** Characteristics of the radionuclides used in this study [9]

Radionuclide	^177^Lu	^161^Tb	^90^Y
Half-life (d)	6.65	6.89	2.67
*β*¯ mean (keV/nt)	133	154	933
Internal conversion/auger (keV/nt)	14.7	48.2	0.2
Total (keV/nt)	148.0	202.5	933.1
# Internal conversion/auger electrons emitted (/nt)	1.27	12.38	1.39E−03
# Internal conversion/auger electrons emitted < 50 keV (/nt)	1.17	12.36	1.27E−03
Photons* (keV)		45.27 (6.6%)	–
	112.95 (6.4%)	46.08 (11.8%)	
	208.36 (11.0%)	48.91 (17.0%)	
		74.57 (10.2%)	

*Photon energies suitable for imaging

**Fig. 1:**
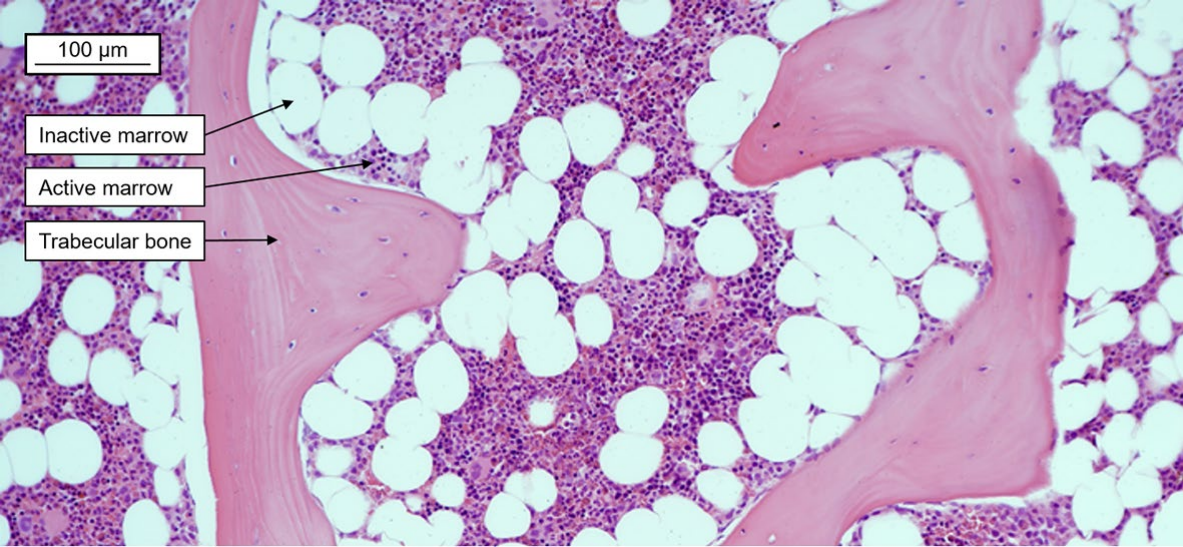
Healthy lumbar spongiosa biopsy from a 65-year-old male showing active and inactive marrow and trabecular bone

To monitor and reduce severe side effects, mean absorbed dose limits were adopted from external beam radiotherapy and iodine-131 treatments for the kidneys and bone marrow at 23–28 Gy and 2 Gy, respectively [20]. The size of the kidneys makes clinical dosimetry fairly straightforward, as the approximation of an absorbed electron energy fraction equal to 1 (*φ *≈ 1) holds for radionuclides currently used in PRRT. In the bone marrow, the dosimetry is challenging, as marrow is contained in small heterogeneous regions in cavities surrounded by trabecular and cortical bone. The bone marrow is further divided into active marrow (AM), which contains stem cells and cells from the hematopoietic progenitor lineage, and inactive marrow (IM), which is comprised of roughly 95% adipocytes (Fig. 1) [21]. Therefore, the electrons cannot be assumed to deposit all energy within the target region of the AM (*φ* < 1). This can be observed by dividing self-irradiation S-values calculated by assuming an absorbed fraction equal to 1 with kidney and bone marrow model S-values (Table 2). The much lower absorbed fraction of the bone marrow model produces large differences for all three radionuclides compared to the kidney model.

**Table 2:** The fraction between rudimentary self-irradiation S-values (calculated by assuming an absorbed fraction equal to 1 and the total energy emission per decay) and model-based self-irradiation S-values from either a kidney model (OLINDA version 2.1) or the bone marrow model used in this study [22]. A fraction differing from 1 emphasizes the complexity of the dosimetry model compared to the rudimentary approach

Radionuclide	Kidney	Bone marrow
^177^Lu	1.00	1.97
^161^Tb	1.01	1.78
^90^Y	1.07	2.84

Clinical bone marrow dosimetry is usually performed using an image-based or blood-based approach [23]. Image-based techniques rely on post-treatment imaging, usually single photon emission computed tomography (SPECT), to quantify uptake in the bone marrow. The blood-based approach uses a ratio to relate activity concentrations determined by blood samples to the activity concentration in the active bone marrow. A correlation between hematological and absorbed dose estimates for 177Lu-DOTATATE has been demonstrated for image-based methods, but no correlation was demonstrated for blood-based dosimetry in a larger prospective study [5, 6, 20, 24].

To improve bone marrow dosimetry, tremendous effort has been put into the development of small-scale skeletal models [25–27]. The irradiation of AM, the surrogate region of the radiosensitive hematopoietic stem and progenitor cells, was evaluated by compartmentalizing the spongiosa region optically scanned or imaged by MRI or micro-CT into AM, IM, and trabecular bone volume and surface (TBV and TBS, respectively). The amount of AM in the marrow space at a specific skeletal site, known as cellularity (Eq. 1), is usually defined in the models through a reference value. However, varying cellularity in patients due to, for example, age and prior treatments will have an impact on the S-values [21].

1 $${\text{Cellularity}} = 1 - \frac{{V_{{{\text{IM}}}} }}{{V_{{{\text{AM}}}} + V_{{{\text{IM}}}} }}$$

where *V*_AM_ and *V*_IM_ are the AM and IM volume, respectively, at a specific skeletal site.

The aim of the present study was to determine useful clinical S-values for terbium-161 and address the potential limitations for patient-specific bone marrow dosimetry with currently available small-scale skeletal models. The estimated S-values for terbium-161 were compared to the corresponding values for lutetium-177 and yttrium-90.

## Methods

### Skeletal dosimetry model

This study was based on data from the male and female skeletal models described by Hough, O’Reilly, and Geyer et al. [28–30]. In these models, the spongiosa bone region is based on micro-CT imaging of multiple bone samples from a 40-year-old male and 45-year-old female and divided into AM, IM, and trabecular bone at a resolution of 50 µm. Trabecular bone could be segmented in the filtered micro-CT images, but the number of voxels corresponding to AM and IM in each cancellous bone sample was determined by the cellularity defined in ICRP 70 [31]. Using the MCNP Monte Carlo program, the authors performed electron transport simulations on both the macrostructure and microstructure of the University of Florida Adult Male/Female Hybrid Phantoms (UFHADM/UFHADF) to calculate specific absorbed fractions (SAFs) by AM targets in a number of skeletal regions [32]. These SAFs were published as supplementary materials and used in this study.

### Model application

**Fig. 2:**
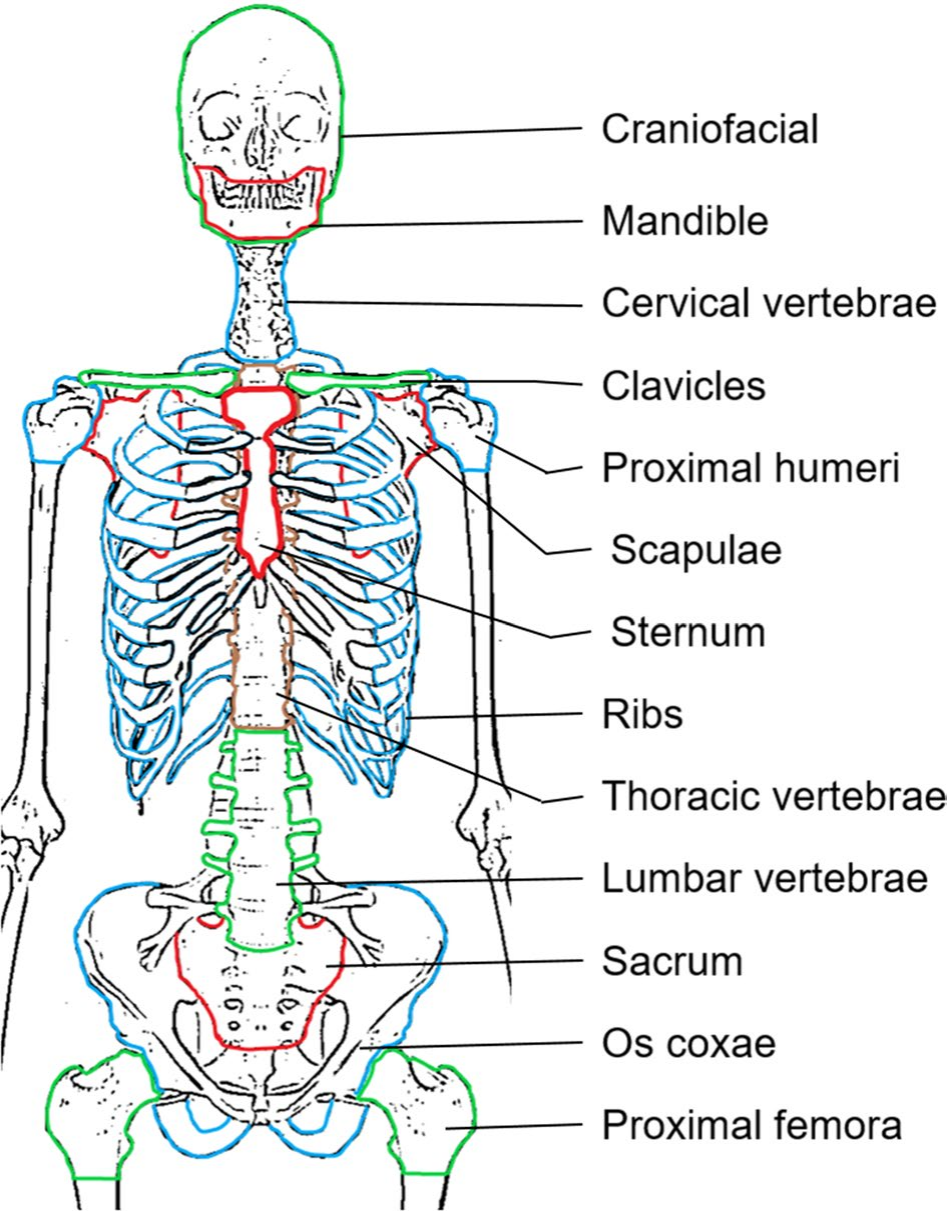
Schematic image of the 13 skeletal sites used to calculate S-values in this study

**Fig. 3:**
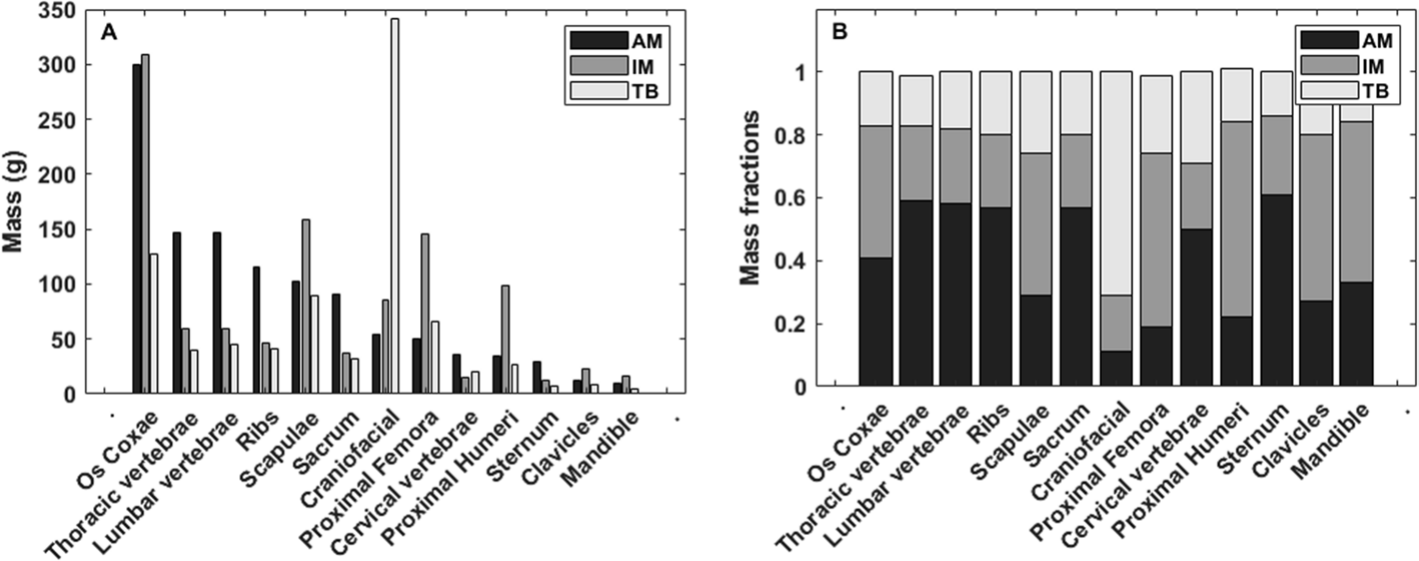
Active marrow, inactive marrow, and trabecular bone masses of the University of Florida Adult Male Hybrid Phantom for 13 skeletal sites **A** and the corresponding mass fractions of each skeletal site **B** [28]

The SAFs were combined with decay data from ICRP 107 to obtain S-values for lutetium-177, terbium-161, and yttrium-90 [9]. The SAFs were tabulated on a fixed monoenergetic electron energy grid ranging from 1 to 10^4^ keV (male) or 10 to 10^4^ keV (female) for the following target/source combinations for the 13 skeletal sites in Fig. 2: (AM ← AM), (AM ← IM), (AM ← TBV), and (AM ← TBS). The skeletal sites differed between the genders, as well as in composition and size (Fig. 3 and Additional file 1: Tables S1, S2) [28, 29]. Similarly, SAFs were provided for self-irradiation [i.e., (AM ← AM)] for bone cavity cellularities varying between 10 and 100%. Decay data included the composite *β*-spectrum, as well as the yields and energies of internal conversion and Auger electrons. Skeletal-averaged S-values were computed using the mass fractions of each skeletal region in the UFHADM/UFHADF (Eq. 2) as derived in Appendix B by Pafundi et al. [33]:

2 $$S_{{{\text{Skeletal }}\;{\text{average}}}} = \mathop \sum \limits_{x} \frac{{m_{{{\text{target}},x}} }}{{m_{{{\text{target}},tot}} }}\frac{{m_{{{\text{source}},x}} }}{{m_{{{\text{source}},tot}} }}S_{x} \left( {{\text{target}} \leftarrow {\text{source}}} \right)$$

where *m*_*x*_ is the target or source mass for skeletal region *x*, *m*_tot_ is the sum of source or target volume for all skeletal regions, and *S*_*x*_ is the S-value calculated for skeletal region *x*. The same methodology can be extended by averaging over the source compartments, resulting in Eq. 3. This is particularly useful, for example, in SPECT quantification when image resolution limits the distinction between trabecular bone and marrow.

3 $$S\left( {{\text{Spongiosa}} \leftarrow {\text{Spongiosa}}} \right) = \mathop \sum \limits_{{{\text{source}}}} \frac{{m_{{{\text{source}}}} }}{{m_{{{\text{Spongiosa}}}} }}S_{{{\text{source}}}} \left( {{\text{Spongiosa}} \leftarrow {\text{source}}} \right)$$

### Monte Carlo simulations to investigate the impact of skeletal model resolution

To highlight the varying impact of a dosimetry model resolution of 50 µm voxels on the three radionuclides included in this study, PENELOPE-2014 was used to simulate energy deposition from a point source in two cubic voxel grids [34]. The characteristics of each point source were described using the PENELOPE subroutine package PENNUC, which enables simulation of nuclear decay [35]. 10^7^ decays were simulated for each point source in a spherical geometry consisting of soft tissue (ICRP). Energy deposition was tallied in cubic voxels emanating radially from the point source with 50-µm voxel sides representing the bone marrow model and 8.2-µm voxel sides corresponding to the average diameter of a hematopoietic stem cell [36].

## Results

### Skeletal site-specific S-values

**Fig. 4:**
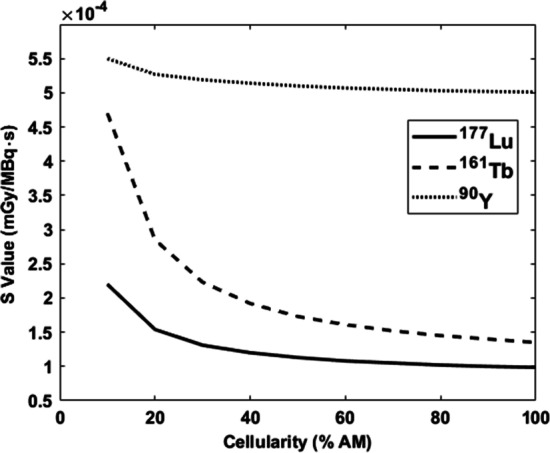
Impact of varying cellularity on S(AM ← AM) in the lumbar vertebrae for the three radionuclides studied. The ICRP 70 reference cellularity is 70% for the lumbar vertebrae

S-values were calculated for the adult male and female phantoms for the 13 skeletal sites in Fig. 2 (Additional file 1: Tables S3, S4). The largest discrepancies between terbium-161 and lutetium-177 (> 60%) arose for AM self-irradiation in bones that contain a minor fraction (< 2%) of AM, such as the mandible and clavicle. This can be explained by less cross-irradiation due to the increased distances between AM voxels due to varying cellularity as visualized in Fig. 4. At skeletal sites containing most of the AM (~ 65%), such as Os coxae, thoracic and lumbar vertebrae, and the ribs, the difference was smaller (< 55%).

### Skeletal-averaged S-values

The S-values in Additional file 1: Tables S3, S4 were averaged using Eq. 2 (Table 3). As expected, S-values decreased as the source/target distance increased. Of all four source distributions, yttrium-90 had the highest S-values of the three radionuclides, roughly 65–80% higher for AM self-irradiation and increasing with the source/target distance. Similarly, all S-values were larger for terbium-161 than for lutetium-177; however, unlike for yttrium-90, these differences decreased as the source was moved away from the AM.

**Table 3:** Skeletal-averaged S-values (mGy/MBq-s) for lutetium-177, terbium-161, and yttrium-90

Radionuclide	S(AM ← AM)		S(AM ← IM)		S(AM ← TBV)		S(AM ← TBS)	
	Female	Male	Female	Male	Female	Male	Female	Male
^177^Lu	1.40E−05	1.07E−05	4.30E−06	2.97E−06	2.27E−06	2.10E−06	5.03E−06	3.66E−06
^161^Tb	2.10E−05	1.61E−05	5.01E−06	3.43E−06	2.70E−06	2.49E−06	6.78E−06	4.93E−06
^90^Y	6.08E−05	4.66E−05	2.34E−05	1.74E−05	2.18E−05	1.98E−05	3.10E−05	2.34E−05

### Spongiosa S-values

In Table 4, S-values for a homogenous distribution of spongiosa activity were calculated using Eq. 3 combined with the skeletal-averaged S-values from the sources AM, IM, and TBS from Table 3. Compared to the skeletal-averaged self-irradiation S-values (S(AM ← AM)), all spongiosa S-values were lower, with the largest effect of the averaging processes seen for terbium-161. Spongiosa S-values for the 13 skeletal sites can be found in Additional file 1: Tables S5 and S6.

**Table 4:** Skeletal-averaged spongiosa S-values (mGy/MBq-s) calculated using the sources AM, IM, and TBS. The fraction from the skeletal average S(AM ← AM) in Table 3 is calculated in the columns on the right

Radionuclide	S(Spongiosa ← Spongiosa)		Fraction from skeletal average S(AM ← AM)	
	Female	Male	Female	Male
^177^Lu	8.21E−06	6.01E−06	0.59	0.56
^161^Tb	1.12E−05	8.57E−06	0.53	0.53
^90^Y	3.99E−05	2.99E−05	0.66	0.64

### Cellularity dependence

The relative impact of varying cellularity (10–100%) on self-irradiation S-values was calculated for the lumbar vertebrae (Fig. 4). For all radionuclides, the S-values initially decreased with increasing fractions of AM, with terbium-161 showing the greatest dependence on cellularity. Similar trends were seen for the other skeletal sites (Additional file 1: Tables S7–S9) except for the craniofacial bone sites were small dimensions and the high-energy electrons of yttrium-90 lower the S-values compared to the other radionuclides.

### Monte Carlo simulations to investigate the impact of skeletal model resolution

**Fig. 5:**
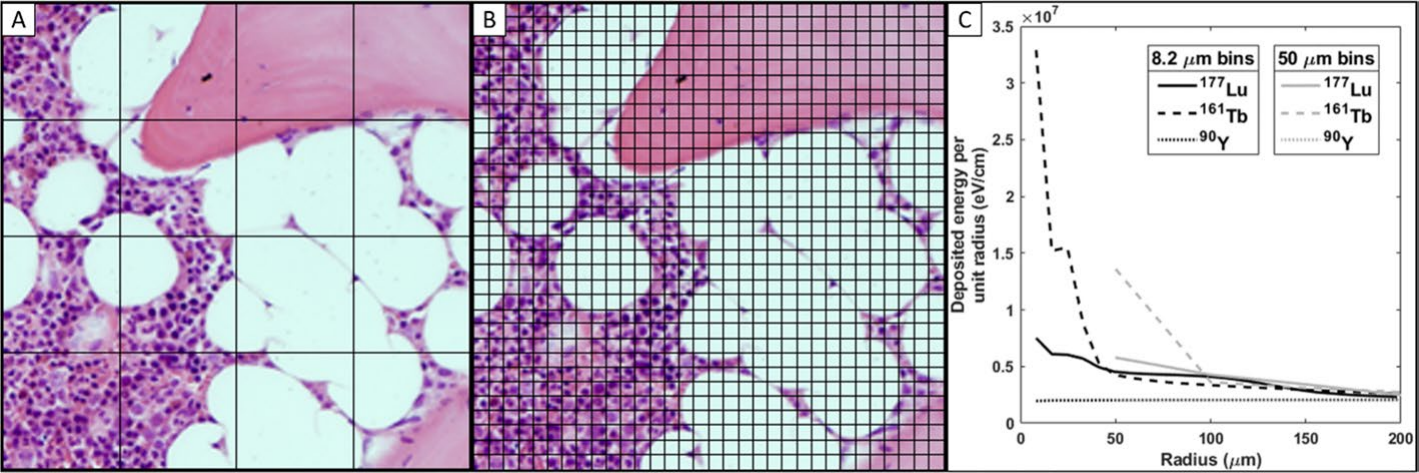
Visualization of two pixel grids corresponding to 50 µm **A** and 8.2 µm **B** on top of a spongiosa biopsy. **C** Point-source energy deposition in the first 200 µm for the three studied radionuclides in a voxel grid of 8.2 µm (solid) and 50 µm (dashed)

In the voxel grid Monte Carlo simulations (Fig. 5C), the steep energy deposition gradients of low-energy electron emitters can be seen clearly within the first 200 µm. Yttrium-90 showed less distance or voxel resolution-dependence than the other two radionuclides.

**Fig. 6:**
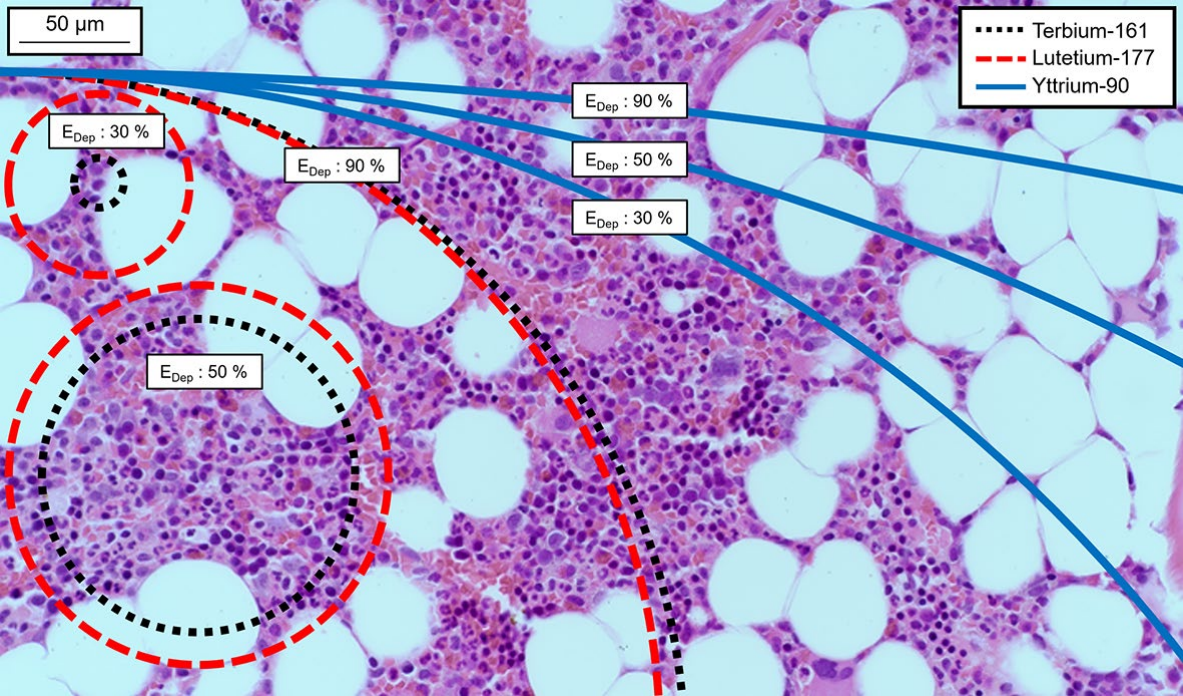
A perspective of the range of energy deposition from a point source on a healthy spongiosa biopsy. 30%, 50%, and 90% of the total emitted electron energy from each of the three radionuclides are deposited within the respective black (terbium-161), red (lutetium-177), and blue circles (yttrium-90). The high energy and long range of yttrium-90 *β*-particles lead to circles with comparatively large radii

The average range of each radionuclide within the spongiosa is demonstrated in Fig. 6, with circles representing 30%, 50%, and 90% energy deposition from a point source. For example, in the figure, the smallest black circle represents the average distance from a point source in which terbium-161 deposits 30% of its total electron energy emission (Table 1), illustrating the contribution of low-energy electrons to the energy deposition of 161-terbium.

## Discussion

In the present study, S-values for terbium-161 were generated for different source distributions using skeletal dosimetry models. Numerous theoretical studies have reported significant local energy deposition of terbium-161, which is suitable when treating small tumors or disseminated disease [10–13]. Hindie et al. performed dose calculations on spheres with diameters of 10–10,000 µm, whereas Bernhardt et al. described the absorbed dose necessary for metastatic control. In both studies, terbium-161 surpassed lutetium-177 and yttrium-90 in terms of the absorbed dose delivered to small tumors. This study focused on the bone marrow as a risk organ and comparisons were made by computing S-values for four source distributions in 13 skeletal sites, as well as the whole-body and homogenous spongiosa average S-values. The study also showed a large source compartment dependence for terbium-161, indicating the importance of increased accuracy in the determination of spongiosa activity distribution when studying low-energy electron emitters.

The 50-µm resolution can be limiting when studying low-energy internal conversion and Auger electron-emitting radionuclides. For example, in a homogenous activity distribution inside a single voxel terbium-161 would deposit 28.2% of the emitted energy in a 50-µm voxel and 14.3% inside the dimensions of a single hematopoietic stem cell (8.2-µm voxel) (Additional file 1: Table S11 and Fig. 5). The corresponding values for lutetium-177 would be 12.9% and 4.8%, and 0.6% and 0.2% for yttrium-90. Consequently, the effects of the source distribution in the spongiosa region will be greater for terbium-161 than for the other two radionuclides. As the source is moved away from the AM target, the impact of low-energy electrons is reduced (Table 3). The source compartment dependence was less evident for long-range electron emitters (e.g., yttrium-90). This is a conceivable reason for the higher hematological toxicity rates observed for [^90^Y]Y-DOTATOC and [^90^Y]Y-DOTATATE compared to [^177^Lu]Lu-DOTATATE and reflected in the higher, less source-dependent S-values for yttrium-90 in this study [1, 7, 37].

To correctly evaluate the risk of hematological toxicity in ^177^Lu- and ^161^Tb-based PRRT, conclusive evidence of the activity distribution inside the bone cavities is needed. The choice between terbium-161 and lutetium-177 would not influence the activity distribution if the same targeting peptide is applied, as recently shown in a preclinical biodistribution study reported by Borgna et al. [15]. Assuming no specific AM uptake, as indicated by the bone marrow aspirations reported by Forrer et al., the irradiation of AM is likely to be highly heterogeneous because there are variations in the blood volume and blood flow inside the bone cavities, as well as an irregular distribution of hematopoietic stem cells in the trabecular bone cavities [38–40]. Such a scenario would presumably reduce AM irradiation from low-energy electrons and could prove advantageous for terbium-161.

Discrepancies have been observed between the absorbed dose in bone marrow calculated by image-based and blood-based dosimetry. Image-based dosimetry has reported threefold or fourfold higher absorbed doses than blood-based methods and established correlations to hematological toxicities [41, 42]. This could indicate active bone marrow uptake related to, for example, the transchelation of lutetium-177 onto transferrin, as discussed recently by Walrand et al. [43]. Such uptake would likely increase the absorbed dose to cells with transferrin receptor expression as the cells are included in the erythroblastic islands [44]. Consequently, these cells will receive increased absorbed doses and the hematological stem cells will receive decreased absorbed doses, especially for low-energy electron emitters such as terbium-161. [45, 46]. The effects of non-specific marrow uptake can be illustrated by spongiosa S-values (Table 4) or by comparing AM self-irradiation with IM, TBV, or TBS sources (Additional file 1: Table S10). These examples indicate an increased sparing effect to AM for terbium-161 compared to lutetium-177 and yttrium-90.

Multiple studies have suggested that age has a significant impact on the induction of hematological toxicity after PRRT [7, 47]. Other factors are equally associated with an increased risk, for example, prior irradiation or chemotherapy, bone metastases, and impaired kidney function [48].

In a study of 324 patients with NETs treated with [^177^Lu]Lu-DOTATATE, Bergsma et al. found a correlation between older age and the severity of the toxicity, which correlates with a decreasing amount of AM, leading to increased S-values (Fig. 4) [49].

As one can expect, [^177^Lu]Lu-DOTATATE-based studies on NETs have shown that the majority of patients are older than the man (40 years old) and woman (45 years old) skeletal-based models. Two studies reported that the mean age and SD of patients were 63 ± 9 (*n* = 229) and 58.1 ± 13.1 (*n* = 42) [50, 51]. The impact of this discrepancy is inherently difficult to quantify, but varying cellularity and its effect on S-values was previously described by Geyer et al., who concluded that AM self-irradiation is significantly affected, especially for low-energy electron emitters [52]. Our results for the lumbar vertebrae (Fig. 4) are in agreement with these observations, as the S-values increase by 110%, 210%, and 9% from reference (70%) for lutetium-177, terbium-161, and yttrium-90, respectively, for 10% cellularity.

In accordance with earlier studies, both histological- and image-based, Salas-Ramirez et al. determined decreasing cellularity with age in the lumbar vertebrae of 44 patients (21 female patients: 19–87 years, 23 male patients: 23–85 years) using MRI [53–55]. The authors observed a faster conversion rate of AM to IM for female patients (0.5% per year) than for male patients (0.3% per year) and a large inter-patient variability of AM differing up to 60% between patients of the same age and gender. Large conversion rates were also seen in a 2014 study by Carmona et al. [56], where 12 patients received external beam radiotherapy and chemotherapy (cisplatin, 5-fluorouracil/mitomycin C [FU/MMC], or cisplatin/5-FU/cetuximab). MR-imaging showed an increase in IM in the vertebral column (T10-S2) of 3.93–10.1% relative to the reference vertebrae (C3-T9).

Given the high age of some of the patients and combination of influencing factors, large active marrow conversions and deviations from the reference cellularity are likely. Such variations could be accounted for by utilizing adaptable skeletal dosimetry models to increase the overall accuracy of bone marrow dosimetry and toxicity correlations. This can be done by quantifying patient cellularity with CT or MRI and calculating S-values using published data for AM self-irradiation from Hough et al. and O’Reilly et al. [28, 29].

## Conclusion

This study showed higher source distribution dependency for terbium-161 compared to lutetium-177 and yttrium-90. Our results indicate the need for a better understanding of the distribution of a respective radiopharmaceutical in the spongiosa bone region to accurately evaluate the risk of AM irradiation from low-energy electron emitters.

## Supplementary Information

**Additional file 1**: **Table S1.** Tissue data from University of Florida Adult Male Hybrid Phantom. **Table S2.** Tissue data from University of Florida Adult Female Hybrid Phantom. **Table S3.** Active marrow S-values (mGy/MBq-s) for yttrium-90, lutetium-177 and terbium-161 for adult male. **Table S4.** Active marrow S-values (mGy/MBq-s) for yttrium-90, lutetium-177 and terbium-161 for adult female. **Table S5.** Skeletal spongiosa S-values (mGy/MBq-s) for adult male calculated using the sources AM, IM and TBS. **Table S6.** Skeletal spongiosa S-values (mGy/MBq-s) for adult female calculated using the sources AM, IM and TBS. **Table S7.** Active marrow S-values (mGy/MBq-s) for varying cellularities for lutetium-177 for adult male. **Table S8.** Active marrow S-values (mGy/MBq-s) for varying cellularities for terbium-161 for adult male. **Table S9.** Active marrow S-values (mGy/MBq-s) for varying cellularities for yttrium-90 for adult male. **Table S10.** Difference (%) between IM, TBV and TBS source distributions and active marrow self-irradiation for skeletal-averaged S-values (**Table S4**) for lutetium-177, terbium-161 and yttrium-90. **Table S11**. Deposited energy (keV) from homogenously distributed activity in two cubic voxels, sized 50 µm and 8.2 µm and the corresponding percent of total emitted energy.

## Data Availability

All data generated or analyzed during this study are included in this published article and its Additional file.

## Abbreviations

NETs: Neuroendocrine tumors
^177^Lu: Lutetium-177
^161^Tb: Terbium-161
^90^Y: Yttrium-90
PRRT: Peptide receptor radiotherapy
AM: Active marrow
IM: Inactive marrow
TBV: Trabecular bone volume
TBS: Trabecular bone surface
S-value: Absorbed dose rate per unit activity
UFHADM: University of Florida adult male hybrid phantom
UFHADF: University of Florida adult female hybrid phantom
