# Supplementary material for: Active bone marrow S-values for the low-energy electron emitter terbium-161 compared to S-values for lutetium-177 and yttrium-90
Source: EJNMMI Phys. 2022 Sep 24;9:65. doi: 10.1186/s40658-022-00495-7 (PMC9509518; doi:10.1186/s40658-022-00495-7)
Supplement: Supplementary file 1 — Additional file 1: Table S1. Tissue data from University of Florida Adult Male Hybrid Phantom. Table S2. Tissue data from University of Florida Adult Female Hybrid Phantom. Table S3. Active marrow S-values (mGy/MBq-s) for yttrium-90, lutetium-177 and terbium-161 for adult male. Table S4. Active marrow S-values (mGy/MBq-s) for yttrium-90, lutetium-177 and terbium-161 for adult female. Table S5. Skeletal spongiosa S-values (mGy/MBq-s) for adult male calculated using the sources AM, IM and TBS. Table S6. Skeletal spongiosa S-values (mGy/MBq-s) for adult female calculated using the sources AM, IM and TBS. Table S7. Active marrow S-values (mGy/MBq-s) for varying cellularities for lutetium-177 for adult male. Table S8. Active marrow S-values (mGy/MBq-s) for varying cellularities for terbium-161 for adult male. Table S9. Active marrow S-values (mGy/MBq-s) for varying cellularities for yttrium-90 for adult male. Table S10. Difference (%) between IM, TBV and TBS source distributions and active marrow self-irradiation for skeletal-averaged S-values (Table S4) for lutetium-177, terbium-161 and yttrium-90. Table S11. Deposited energy (keV) from homogenously distributed activity in two cubic voxels, sized 50 µm and 8.2 µm and the corresponding percent of total emitted energy. [file 40658_2022_495_MOESM1_ESM.docx]

SUPPLEMENTAL TABLE 1. Tissue data from University of Florida Adult Male Hybrid Phantom

| **Skeletal site** | **Tissue mass (g)** | | | **Tissue fraction** | | |
| --- | --- | --- | --- | --- | --- | --- |
|  | **AM** | **IM** | **TB** | **fAM** | **fIM** | **fTB** |
| Craniofacial | 54.8 | 85.1 | 341.5 | 0.11 | 0.18 | 0.71 |
| Mandible | 10.1 | 15.7 | 4.8 | 0.33 | 0.51 | 0.16 |
| Scapulae | 102.3 | 158.9 | 89.7 | 0.29 | 0.45 | 0.26 |
| Clavicles | 12.0 | 23.2 | 8.8 | 0.27 | 0.53 | 0.20 |
| Sternum | 29.4 | 12.0 | 6.9 | 0.61 | 0.25 | 0.14 |
| Ribs | 115.0 | 46.9 | 41.1 | 0.57 | 0.23 | 0.20 |
| Cervical vertebrae | 36.1 | 14.7 | 20.7 | 0.50 | 0.21 | 0.29 |
| Thoracic vertebrae | 147.2 | 60.0 | 40.4 | 0.59 | 0.24 | 0.16 |
| Lumbar vertebrae | 146.9 | 59.9 | 44.9 | 0.58 | 0.24 | 0.18 |
| Sacrum | 91.4 | 37.3 | 32.2 | 0.57 | 0.23 | 0.20 |
| Os Coxae | 299.7 | 308.9 | 127.2 | 0.41 | 0.42 | 0.17 |
| Proximal Humeri | 34.8 | 99.2 | 27.2 | 0.22 | 0.62 | 0.17 |
| Proximal Femora | 50.9 | 145.2 | 66.1 | 0.19 | 0.55 | 0.25 |
| Total | 1130.6 | 1067.0 | 851.5 | 0.37 | 0.35 | 0.28 |

SUPPLEMENTAL TABLE 2. Tissue data from University of Florida Adult Female Hybrid Phantom

| **Skeletal site** | **Tissue mass (g)** | | | **Tissue fraction** | | |
| --- | --- | --- | --- | --- | --- | --- |
|  | **AM** | **IM** | **TB** | **fAM** | **fIM** | **fTB** |
| Craniofacial | 29.1 | 45 | 303 | 0.08 | 0.12 | 0.80 |
| Mandible | 7.7 | 12 | 6.7 | 0.29 | 0.45 | 0.25 |
| Scapulae | 66.8 | 103.6 | 18.6 | 0.35 | 0.55 | 0.10 |
| Clavicles | 8.8 | 16.9 | 4.2 | 0.29 | 0.57 | 0.14 |
| Sternum | 18.4 | 7.5 | 4.4 | 0.61 | 0.25 | 0.15 |
| Ribs | 112.1 | 45.6 | 29.8 | 0.60 | 0.24 | 0.16 |
| Cervical vertebrae | 29.5 | 12 | 17.1 | 0.50 | 0.20 | 0.29 |
| Thoracic vertebrae | 112.2 | 45.7 | 38.5 | 0.57 | 0.23 | 0.20 |
| Lumbar vertebrae | 140.7 | 57.3 | 54.2 | 0.56 | 0.23 | 0.21 |
| Sacrum | 58.8 | 60.5 | 40.5 | 0.37 | 0.38 | 0.25 |
| Os Coxae | 224.9 | 231.5 | 42.5 | 0.45 | 0.46 | 0.09 |
| Proximal Humeri | 35.5 | 62.7 | 19.1 | 0.30 | 0.53 | 0.16 |
| Proximal Femora | 44.4 | 78.2 | 68.8 | 0.23 | 0.41 | 0.36 |
| Tot | 888.9 | 778.5 | 647.4 | 0.38 | 0.34 | 0.28 |

SUPPLEMENTAL TABLE 3. Active marrow S values (mGy/MBq-s) for yttrium-90, lutetium-177 and terbium-161 for adult male

| **Skeletal site** | **S(AM ← AM)** | | | **S(AM ← IM)** | | | **S(AM ← TBV)** | | | **S(AM ← TBS)** | | |
| --- | --- | --- | --- | --- | --- | --- | --- | --- | --- | --- | --- | --- |
|  | **^90^Y** | **^177^Lu** | **^161^Tb** | **^90^Y** | **^177^Lu** | **^161^Tb** | **^90^Y** | **^177^Lu** | **^161^Tb** | **^90^Y** | **^177^Lu** | **^161^Tb** |
| Craniofacial | 3.57E-04 | 1.39E-04 | 2.42E-04 | 3.34E-04 | 7.66E-05 | 8.82E-05 | 1.89E-04 | 2.07E-05 | 2.45E-05 | 2.78E-04 | 6.45E-05 | 9.12E-05 |
| Mandible | 3.67E-03 | 9.66E-04 | 1.56E-03 | 3.59E-03 | 6.92E-04 | 7.97E-04 | 3.24E-03 | 3.53E-04 | 4.18E-04 | 3.37E-03 | 5.38E-04 | 7.19E-04 |
| Scapulae | 3.69E-04 | 9.31E-05 | 1.52E-04 | 3.66E-04 | 6.74E-05 | 7.77E-05 | 3.16E-04 | 2.88E-05 | 3.42E-05 | 3.39E-04 | 5.05E-05 | 6.79E-05 |
| Clavicles | 2.69E-03 | 7.28E-04 | 1.22E-03 | 2.62E-03 | 4.96E-04 | 5.71E-04 | 2.16E-03 | 2.12E-04 | 2.52E-04 | 2.27E-03 | 3.52E-04 | 4.77E-04 |
| Sternum | 2.45E-03 | 5.27E-04 | 7.62E-04 | 2.44E-03 | 4.42E-04 | 5.10E-04 | 2.28E-03 | 2.65E-04 | 3.12E-04 | 2.33E-03 | 3.54E-04 | 4.71E-04 |
| Ribs | 4.39E-04 | 1.28E-04 | 1.86E-04 | 4.31E-04 | 1.04E-04 | 1.20E-04 | 3.99E-04 | 5.61E-05 | 6.62E-05 | 4.10E-04 | 8.19E-05 | 1.10E-04 |
| Cervical vertebrae | 1.56E-03 | 3.95E-04 | 5.79E-04 | 1.54E-03 | 3.17E-04 | 3.65E-04 | 1.44E-03 | 1.72E-04 | 2.04E-04 | 1.48E-03 | 2.54E-04 | 3.43E-04 |
| Thoracic vertebrae | 4.84E-04 | 1.05E-04 | 1.52E-04 | 4.78E-04 | 8.62E-05 | 9.95E-05 | 4.41E-04 | 4.84E-05 | 5.73E-05 | 4.48E-04 | 6.82E-05 | 9.12E-05 |
| Lumbar vertebrae | 5.05E-04 | 1.05E-04 | 1.52E-04 | 4.99E-04 | 8.61E-05 | 9.93E-05 | 4.69E-04 | 4.83E-05 | 5.72E-05 | 4.80E-04 | 6.86E-05 | 9.18E-05 |
| Sacrum | 8.15E-04 | 1.68E-04 | 2.43E-04 | 8.06E-04 | 1.38E-04 | 1.59E-04 | 7.57E-04 | 7.35E-05 | 8.74E-05 | 7.77E-04 | 1.09E-04 | 1.46E-04 |
| Os Coxae | 1.81E-04 | 3.85E-05 | 5.95E-05 | 1.78E-04 | 2.92E-05 | 3.37E-05 | 1.68E-04 | 1.75E-05 | 2.07E-05 | 1.70E-04 | 2.37E-05 | 3.16E-05 |
| Proximal Humeri | 8.74E-04 | 2.16E-04 | 3.79E-04 | 7.99E-04 | 1.34E-04 | 1.54E-04 | 7.56E-04 | 6.96E-05 | 8.29E-05 | 7.77E-04 | 1.02E-04 | 1.37E-04 |
| Proximal Femora | 5.41E-04 | 1.42E-04 | 2.53E-04 | 5.19E-04 | 8.69E-05 | 1.00E-04 | 4.57E-04 | 4.46E-05 | 5.30E-05 | 4.79E-04 | 6.75E-05 | 9.07E-05 |

SUPPLEMENTAL TABLE 4. Active marrow S values (mGy/MBq-s) for yttrium-90, lutetium-177 and terbium-161 for adult female

| **Skeletal site** | **S(AM ← AM)** | | | **S(AM ← IM)** | | | **S(AM ← TBV)** | | | **S(AM ← TBS)** | | |
| --- | --- | --- | --- | --- | --- | --- | --- | --- | --- | --- | --- | --- |
|  | **^90^Y** | **^177^Lu** | **^161^Tb** | **^90^Y** | **^177^Lu** | **^161^Tb** | **^90^Y** | **^177^Lu** | **^161^Tb** | **^90^Y** | **^177^Lu** | **^161^Tb** |
| Craniofacial | 4.79E-04 | 2.31E-04 | 4.15E-04 | 4.55E-04 | 1.47E-04 | 1.69E-04 | 2.82E-04 | 3.37E-05 | 3.98E-05 | 4.29E-04 | 1.15E-04 | 1.64E-04 |
| Mandible | 4.37E-03 | 1.21E-03 | 1.96E-03 | 4.28E-03 | 9.05E-04 | 1.05E-03 | 3.58E-03 | 2.91E-04 | 3.51E-04 | 3.89E-03 | 6.25E-04 | 8.47E-04 |
| Scapulae | 5.92E-04 | 1.44E-04 | 2.31E-04 | 5.82E-04 | 1.08E-04 | 1.26E-04 | 5.58E-04 | 6.86E-05 | 8.07E-05 | 5.69E-04 | 9.21E-05 | 1.21E-04 |
| Clavicles | 3.83E-03 | 9.92E-04 | 1.64E-03 | 3.76E-03 | 7.21E-04 | 8.40E-04 | 3.34E-03 | 3.27E-04 | 3.90E-04 | 3.52E-03 | 5.51E-04 | 7.35E-04 |
| Sternum | 3.86E-03 | 8.52E-04 | 1.23E-03 | 3.82E-03 | 7.24E-04 | 8.44E-04 | 3.41E-03 | 3.09E-04 | 3.70E-04 | 3.61E-03 | 5.57E-04 | 7.44E-04 |
| Ribs | 4.48E-04 | 1.34E-04 | 1.94E-04 | 4.43E-04 | 1.13E-04 | 1.31E-04 | 3.96E-04 | 4.86E-05 | 5.77E-05 | 4.22E-04 | 8.86E-05 | 1.18E-04 |
| Cervical vertebrae | 1.91E-03 | 4.82E-04 | 7.05E-04 | 1.88E-03 | 4.02E-04 | 4.68E-04 | 1.73E-03 | 2.05E-04 | 2.44E-04 | 1.81E-03 | 3.22E-04 | 4.33E-04 |
| Thoracic vertebrae | 6.07E-04 | 1.35E-04 | 1.95E-04 | 6.01E-04 | 1.14E-04 | 1.32E-04 | 5.59E-04 | 6.01E-05 | 7.14E-05 | 5.82E-04 | 9.12E-05 | 1.22E-04 |
| Lumbar vertebrae | 5.18E-04 | 1.08E-04 | 1.56E-04 | 5.13E-04 | 9.12E-05 | 1.06E-04 | 4.69E-04 | 4.45E-05 | 5.30E-05 | 4.92E-04 | 7.18E-05 | 9.61E-05 |
| Sacrum | 8.21E-04 | 1.88E-04 | 2.91E-04 | 8.09E-04 | 1.47E-04 | 1.71E-04 | 7.51E-04 | 7.18E-05 | 8.56E-05 | 7.78E-04 | 1.15E-04 | 1.55E-04 |
| Os Coxae | 2.57E-04 | 5.26E-05 | 8.04E-05 | 2.54E-04 | 4.20E-05 | 4.90E-05 | 2.39E-04 | 2.36E-05 | 2.79E-05 | 2.46E-04 | 3.40E-05 | 4.51E-05 |
| Proximal Humeri | 1.17E-03 | 2.55E-04 | 4.17E-04 | 1.15E-03 | 1.87E-04 | 2.18E-04 | 1.07E-03 | 1.05E-04 | 1.25E-04 | 1.10E-03 | 1.50E-04 | 2.00E-04 |
| Proximal Femora | 7.09E-04 | 2.03E-04 | 3.71E-04 | 6.86E-04 | 1.27E-04 | 1.47E-04 | 6.80E-04 | 6.26E-05 | 7.46E-05 | 7.28E-04 | 1.13E-04 | 1.54E-04 |

SUPPLEMENTAL TABLE 5. Skeletal spongiosa S values (mGy/MBq-s) for adult male calculated using the sources AM, IM and TBS

| **Skeletal site** | **S(Spongiosa ← Spongiosa)** | | |
| --- | --- | --- | --- |
|  | **^90^Y** | **^177^Lu** | **^161^Tb** |
| Craniofacial | 2.97E-04 | 7.52E-05 | 1.08E-04 |
| Mandible | 3.58E-03 | 7.59E-04 | 1.04E-03 |
| Scapulae | 3.60E-04 | 7.06E-05 | 9.67E-05 |
| Clavicles | 2.57E-03 | 5.30E-04 | 7.28E-04 |
| Sternum | 2.43E-03 | 4.81E-04 | 6.58E-04 |
| Ribs | 4.31E-04 | 1.13E-04 | 1.56E-04 |
| Cervical vertebrae | 1.53E-03 | 3.38E-04 | 4.67E-04 |
| Thoracic vertebrae | 4.77E-04 | 9.44E-05 | 1.29E-04 |
| Lumbar vertebrae | 4.99E-04 | 9.40E-05 | 1.29E-04 |
| Sacrum | 8.05E-04 | 1.49E-04 | 2.04E-04 |
| Os Coxae | 1.78E-04 | 3.20E-05 | 4.39E-05 |
| Proximal Humeri | 8.11E-04 | 1.46E-04 | 2.00E-04 |
| Proximal Femora | 5.13E-04 | 9.27E-05 | 1.27E-04 |
| Skeletal average | 2.99E-05 | 6.01E-06 | 8.57E-06 |

SUPPLEMENTAL TABLE 6. Skeletal spongiosa S values (mGy/MBq-s) for adult female calculated using the sources AM, IM and TBS

| **Skeletal site** | **S(Spongiosa ← Spongiosa)** | | |
| --- | --- | --- | --- |
|  | **^90^Y** | **^177^Lu** | **^161^Tb** |
| Craniofacial | 4.36E-04 | 1.28E-04 | 1.84E-04 |
| Mandible | 4.21E-03 | 9.23E-04 | 1.26E-03 |
| Scapulae | 5.84E-04 | 1.19E-04 | 1.63E-04 |
| Clavicles | 3.75E-03 | 7.77E-04 | 1.06E-03 |
| Sternum | 3.81E-03 | 7.78E-04 | 1.06E-03 |
| Ribs | 4.43E-04 | 1.22E-04 | 1.67E-04 |
| Cervical vertebrae | 1.87E-03 | 4.19E-04 | 5.77E-04 |
| Thoracic vertebrae | 6.01E-04 | 1.21E-04 | 1.66E-04 |
| Lumbar vertebrae | 5.11E-04 | 9.65E-05 | 1.32E-04 |
| Sacrum | 8.05E-04 | 1.54E-04 | 2.11E-04 |
| Os Coxae | 2.55E-04 | 4.61E-05 | 6.28E-05 |
| Proximal Humeri | 1.15E-03 | 2.14E-04 | 3.06E-04 |
| Proximal Femora | 7.00E-04 | 1.48E-04 | 2.16E-04 |
| Skeletal average | 3.99E-05 | 8.21E-06 | 1.16E-05 |

SUPPLEMENTAL TABLE 7. Active marrow S values (mGy/MBq-s) for varying cellularities for lutetium-177 for adult male.

| **Lutetium-177** | **S(AM ← AM)** | | | | | | | | | |
| --- | --- | --- | --- | --- | --- | --- | --- | --- | --- | --- |
|  | **Cellularity** | | | | | | | | | |
| **Skeletal site** | **10** | **20** | **30** | **40** | **50** | **60** | **70** | **80** | **90** | **100** |
| Craniofacial | 2.83E-04 | 1.86E-04 | 1.53E-04 | 1.37E-04 | 1.27E-04 | 1.20E-04 | 1.15E-04 | 1.12E-04 | 1.09E-04 | 1.07E-04 |
| Mandible | 1.74E-03 | 1.22E-03 | 1.04E-03 | 9.52E-04 | 8.98E-04 | 8.61E-04 | 8.34E-04 | 8.13E-04 | 7.97E-04 | 7.83E-04 |
| Scapulae | 1.70E-04 | 1.18E-04 | 1.00E-04 | 9.18E-05 | 8.64E-05 | 8.29E-05 | 8.03E-05 | 7.82E-05 | 7.66E-05 | 7.54E-05 |
| Clavicles | 1.27E-03 | 8.82E-04 | 7.51E-04 | 6.86E-04 | 6.46E-04 | 6.19E-04 | 6.00E-04 | 5.85E-04 | 5.74E-04 | 5.64E-04 |
| Sternum | 1.11E-03 | 7.70E-04 | 6.58E-04 | 6.01E-04 | 5.67E-04 | 5.44E-04 | 5.27E-04 | 5.14E-04 | 5.04E-04 | 4.95E-04 |
| Ribs | 2.75E-04 | 1.90E-04 | 1.61E-04 | 1.47E-04 | 1.38E-04 | 1.32E-04 | 1.28E-04 | 1.25E-04 | 1.22E-04 | 1.20E-04 |
| Cervical vertebrae | 8.63E-04 | 5.91E-04 | 5.00E-04 | 4.54E-04 | 4.27E-04 | 4.08E-04 | 3.95E-04 | 3.84E-04 | 3.76E-04 | 3.70E-04 |
| Thoracic vertebrae | 2.20E-04 | 1.53E-04 | 1.31E-04 | 1.20E-04 | 1.13E-04 | 1.08E-04 | 1.05E-04 | 1.02E-04 | 1.00E-04 | 9.87E-05 |
| Lumbar vertebrae | 2.20E-04 | 1.54E-04 | 1.31E-04 | 1.20E-04 | 1.13E-04 | 1.08E-04 | 1.05E-04 | 1.02E-04 | 1.00E-04 | 9.86E-05 |
| Sacrum | 3.53E-04 | 2.46E-04 | 2.10E-04 | 1.92E-04 | 1.81E-04 | 1.73E-04 | 1.68E-04 | 1.64E-04 | 1.60E-04 | 1.58E-04 |
| Os Coxae | 7.43E-05 | 5.18E-05 | 4.42E-05 | 4.04E-05 | 3.81E-05 | 3.65E-05 | 3.54E-05 | 3.45E-05 | 3.38E-05 | 3.33E-05 |
| Proximal Humeri | 3.37E-04 | 2.36E-04 | 2.02E-04 | 1.85E-04 | 1.74E-04 | 1.67E-04 | 1.62E-04 | 1.58E-04 | 1.55E-04 | 1.53E-04 |
| Proximal Femora | 2.25E-04 | 1.56E-04 | 1.33E-04 | 1.21E-04 | 1.14E-04 | 1.09E-04 | 1.06E-04 | 1.03E-04 | 1.01E-04 | 9.92E-05 |

SUPPLEMENTAL TABLE 8. Active marrow S values (mGy/MBq-s) for varying cellularities for terbium-161 for adult male.

| **Terbium-161** | **S(AM ← AM)** | | | | | | | | | |
| --- | --- | --- | --- | --- | --- | --- | --- | --- | --- | --- |
|  | **Cellularity** | | | | | | | | | |
| **Skeletal site** | **10** | **20** | **30** | **40** | **50** | **60** | **70** | **80** | **90** | **100** |
| Craniofacial | 6.40E-04 | 3.70E-04 | 2.80E-04 | 2.35E-04 | 2.08E-04 | 1.90E-04 | 1.77E-04 | 1.67E-04 | 1.59E-04 | 1.53E-04 |
| Mandible | 3.72E-03 | 2.26E-03 | 1.77E-03 | 1.52E-03 | 1.38E-03 | 1.28E-03 | 1.21E-03 | 1.15E-03 | 1.11E-03 | 1.07E-03 |
| Scapulae | 3.65E-04 | 2.20E-04 | 1.72E-04 | 1.48E-04 | 1.33E-04 | 1.24E-04 | 1.17E-04 | 1.11E-04 | 1.07E-04 | 1.04E-04 |
| Clavicles | 2.71E-03 | 1.64E-03 | 1.28E-03 | 1.10E-03 | 9.94E-04 | 9.21E-04 | 8.70E-04 | 8.30E-04 | 8.00E-04 | 7.74E-04 |
| Sternum | 2.36E-03 | 1.43E-03 | 1.12E-03 | 9.63E-04 | 8.70E-04 | 8.07E-04 | 7.62E-04 | 7.28E-04 | 7.02E-04 | 6.80E-04 |
| Ribs | 5.92E-04 | 3.56E-04 | 2.77E-04 | 2.38E-04 | 2.14E-04 | 1.98E-04 | 1.86E-04 | 1.78E-04 | 1.71E-04 | 1.66E-04 |
| Cervical vertebrae | 1.87E-03 | 1.12E-03 | 8.67E-04 | 7.42E-04 | 6.66E-04 | 6.16E-04 | 5.79E-04 | 5.51E-04 | 5.30E-04 | 5.13E-04 |
| Thoracic vertebrae | 4.70E-04 | 2.85E-04 | 2.23E-04 | 1.92E-04 | 1.73E-04 | 1.61E-04 | 1.52E-04 | 1.45E-04 | 1.40E-04 | 1.35E-04 |
| Lumbar vertebrae | 4.70E-04 | 2.85E-04 | 2.23E-04 | 1.92E-04 | 1.73E-04 | 1.61E-04 | 1.52E-04 | 1.45E-04 | 1.40E-04 | 1.35E-04 |
| Sacrum | 7.55E-04 | 4.57E-04 | 3.58E-04 | 3.08E-04 | 2.78E-04 | 2.58E-04 | 2.43E-04 | 2.32E-04 | 2.24E-04 | 2.17E-04 |
| Os Coxae | 1.58E-04 | 9.60E-05 | 7.51E-05 | 6.48E-05 | 5.85E-05 | 5.42E-05 | 5.12E-05 | 4.89E-05 | 4.71E-05 | 4.57E-05 |
| Proximal Humeri | 7.15E-04 | 4.35E-04 | 3.42E-04 | 2.95E-04 | 2.67E-04 | 2.48E-04 | 2.34E-04 | 2.24E-04 | 2.15E-04 | 2.09E-04 |
| Proximal Femora | 4.82E-04 | 2.91E-04 | 2.27E-04 | 1.95E-04 | 1.76E-04 | 1.63E-04 | 1.54E-04 | 1.47E-04 | 1.41E-04 | 1.37E-04 |

SUPPLEMENTAL TABLE 9. Active marrow S values (mGy/MBq-s) for varying cellularities for yttrium-90 for adult male.

| **Yttrium-90** | **S(AM ← AM)** | | | | | | | | | |
| --- | --- | --- | --- | --- | --- | --- | --- | --- | --- | --- |
|  | **Cellularity** | | | | | | | | | |
| **Skeletal site** | **10** | **20** | **30** | **40** | **50** | **60** | **70** | **80** | **90** | **100** |
| Craniofacial | 4.06E-04 | 3.73E-04 | 3.62E-04 | 3.56E-04 | 3.52E-04 | 3.49E-04 | 3.47E-04 | 3.46E-04 | 3.45E-04 | 3.45E-04 |
| Mandible | 3.95E-03 | 3.77E-03 | 3.71E-03 | 3.67E-03 | 3.65E-03 | 3.64E-03 | 3.62E-03 | 3.60E-03 | 3.59E-03 | 3.59E-03 |
| Scapulae | 3.96E-04 | 3.78E-04 | 3.72E-04 | 3.69E-04 | 3.65E-04 | 3.64E-04 | 3.62E-04 | 3.60E-04 | 3.60E-04 | 3.60E-04 |
| Clavicles | 2.88E-03 | 2.76E-03 | 2.70E-03 | 2.67E-03 | 2.66E-03 | 2.65E-03 | 2.63E-03 | 2.62E-03 | 2.61E-03 | 2.61E-03 |
| Sternum | 2.66E-03 | 2.55E-03 | 2.51E-03 | 2.49E-03 | 2.47E-03 | 2.46E-03 | 2.45E-03 | 2.45E-03 | 2.43E-03 | 2.42E-03 |
| Ribs | 4.91E-04 | 4.61E-04 | 4.52E-04 | 4.47E-04 | 4.44E-04 | 4.42E-04 | 4.39E-04 | 4.37E-04 | 4.36E-04 | 4.35E-04 |
| Cervical vertebrae | 1.74E-03 | 1.65E-03 | 1.61E-03 | 1.59E-03 | 1.58E-03 | 1.57E-03 | 1.56E-03 | 1.56E-03 | 1.55E-03 | 1.55E-03 |
| Thoracic vertebrae | 5.28E-04 | 5.05E-04 | 4.97E-04 | 4.92E-04 | 4.89E-04 | 4.87E-04 | 4.84E-04 | 4.82E-04 | 4.81E-04 | 4.80E-04 |
| Lumbar vertebrae | 5.50E-04 | 5.27E-04 | 5.19E-04 | 5.14E-04 | 5.10E-04 | 5.07E-04 | 5.05E-04 | 5.03E-04 | 5.02E-04 | 5.01E-04 |
| Sacrum | 8.89E-04 | 8.52E-04 | 8.37E-04 | 8.29E-04 | 8.24E-04 | 8.20E-04 | 8.15E-04 | 8.12E-04 | 8.09E-04 | 8.06E-04 |
| Os Coxae | 1.94E-04 | 1.86E-04 | 1.83E-04 | 1.82E-04 | 1.80E-04 | 1.80E-04 | 1.79E-04 | 1.78E-04 | 1.78E-04 | 1.77E-04 |
| Proximal Humeri | 9.18E-04 | 8.83E-04 | 8.68E-04 | 8.61E-04 | 8.55E-04 | 8.51E-04 | 8.48E-04 | 8.45E-04 | 8.41E-04 | 8.37E-04 |
| Proximal Femora | 5.71E-04 | 5.46E-04 | 5.37E-04 | 5.32E-04 | 5.29E-04 | 5.26E-04 | 5.24E-04 | 5.22E-04 | 5.20E-04 | 5.19E-04 |

SUPPLEMENTAL TABLE 10. Difference (%) between IM, TBV and TBS source distributions and active marrow self-irradiation for skeletal averaged S values (Table 4) for lutetium-177, terbium-161 and yttrium-90.

| **Radionuclide** | **S_Avg_(AM ← IM)/**  **S_Avg_(AM ← AM)** | | **S_Avg_(AM ← TBV)/**  **S_Avg_(AM ← AM)** | | **S_Avg_(AM ← TBS)/**  **S_Avg_(AM ← AM)** | |
| --- | --- | --- | --- | --- | --- | --- |
|  | **Female** | **Male** | **Female** | **Male** | **Female** | **Male** |
| **177-Lu** | 30.8 | 27.9 | 16.3 | 19.7 | 36.1 | 34.3 |
| **161-Tb** | 23.8 | 21.2 | 12.9 | 15.4 | 24.0 | 30.5 |
| **90-Y** | 38.6 | 37.3 | 35.8 | 42.4 | 51.0 | 50.1 |

SUPPLEMENTAL TABLE 11. Deposited energy (keV) from homogenously distributed activity in two cubic voxels, sized 50 µm and 8.2 µm and the corresponding percent of total emitted energy.

| **Deposited energy (keV)** | ^177^Lu | ^161^Tb | ^90^Y |
| --- | --- | --- | --- |
| 50 µm | 19.1 | 57.1 | 6.1 |
| 8.2 µm | 7 | 29.1 | 1.9 |
| % of total emitted energy | 12.9 | 28.2 | 0.6 |
|  | 4.8 | 14.3 | 0.2 |
